# Women’s bodily experience of antenatal breastmilk expression from 34 weeks of gestation: Qualitative results from the Express-MOM study

**DOI:** 10.18332/ejm/193601

**Published:** 2024-11-01

**Authors:** Sarah Bjerrum Bentzen, Marie Bendix Simonsen, Gitte Zachariassen, Christina Anne Vinter, Kristina Garne Holm

**Affiliations:** 1 Hans Christian Andersen Children’s Hospital, Odense University Hospital, Odense, Denmark; 2 Department of Gynaecology and Obstetrics, Odense University Hospital, Odense, Denmark; 3 Department for Clinical Research, Faculty of Health Sciences, University of Southern Denmark, Odense, Denmark; 4 Department of Paediatrics and Adolescent Medicine, Lillebaelt Hospital-University Hospital of Southern Denmark, Kolding, Denmark; 5 Steno Diabetes Center Odense, Odense University Hospital, Odense, Denmark

**Keywords:** qualitative research, pregnancy, body image, breastfeeding, experience, antenatal breastmilk expression

## Abstract

**INTRODUCTION:**

Breastfeeding establishment can be challenging due to several factors including women’s emotional and physical perception of breastfeeding. This study examines the bodily experiences of healthy women performing antenatal breastmilk expression (aBME) from gestational week 34 until term and whether aBME can support women during breastfeeding establishment.

**METHODS:**

A qualitative phenological-hermeneutic approach was applied. In-depth semi-structured interviews were conducted from December 2022 to March 2023, and women were recruited from the Express-MOM Study, which examined aBME before term. Interviews were conducted 2–4 weeks after birth. Questions concerned body image, bodily experience of aBME, and whether aBME supported their confidence during breastfeeding establishment. Interviews were audio recorded, verbatim transcribed, coded by the first and last author, and subjected to thematic analysis.

**RESULTS:**

Seven women participated in the interviews. Three themes were identified: 1) A desire to be prepared, which identified how women had a deep-felt wish to provide their infants with their milk; 2) Bodily confident, which covered how women trusted their body when expressing milk through aBME, and how this increased their confidence in breastfeeding; and 3) Being confident in the storm, which addressed how knowledge about women’s own body obtained from aBME was valuable in the vulnerable breastfeeding establishment.

**CONCLUSIONS:**

aBME from 34 weeks of gestation can contribute to women feeling more bodily confident and prepared for breastfeeding establishment. This study provides a basis for future research on aBME in women at risk of breastfeeding difficulties (e.g. preterm delivery) to identify if aBME can increase breastfeeding confidence and also breastfeeding initiation and rates.

**CLINICAL TRIAL REGISTRATION:**

The study is registered on the official website of ClinicalTrials.gov

**IDENTIFIER:**

ID NCT05516199

## INTRODUCTION

The World Health Organization (WHO) recommends exclusive breastfeeding for the first six months of an infant’s life. However, only 38% of all infants globally are exclusively breastfed for six months^1^. In Denmark, 79% of infants are exclusively breastfed during the first month, and only 11.6% at six months of age^2,3^. According to the Danish National Children’s Database (BDB) there has been a reduction from 2018 to 2020 in children that are being exclusively breastfed^2^. In preterm infants, the same trend has been reported^4^. Therefore, WHO has set a new global target for the year 2025 that 50% of all infants should be exclusively breastfed for six months^1^. To accomplish the goal, breastfeeding needs to be a public health issue that requires a holistic approach^5^. Evidence on the long-term benefits of breastfeeding is well documented for both mother and infant^6^. However, several factors can influence breastfeeding duration. Sociodemographic factors among mothers such as ethnicity, higher level of education, current job situation, cohabiting are positively associated with breastfeeding duration^2,6^; but also psychological factors such as knowledge, expectations, and confidence in own abilities^6^. In addition, body image concerns in relation to breastfeeding, are also known to influence breastfeeding duration^5,7^. Breastfeeding is a physical experience, and research indicates that breastfeeding also affects women’s thoughts and feelings about their bodies^8^. If breastfeeding mothers were unable to appreciate body changes during pregnancy or were having negative body image concerns, it may lead to negative thoughts, a feeling of sadness, or reduced self-esteem^6^. The study by Broers et al.^9^ revealed that more evidence should focus on the emotional and social context of breastfeeding.

Another factor that can affect women’s thoughts about their bodies is pumping breastmilk. Pumping breastmilk is necessary when delivering preterm and wishing to tube feed the infant with mother’s own milk, and to establish breastfeeding. After birth pumping of milk is also beneficial in women with diabetes, both in terms of their milk supply and their offspring^10^. Until recently, research about antenatal breastmilk expression (aBME) has focused on pregnant women with diabetes and their offspring with the aim of preventing hypoglycemia in the newborn^11^. Furthermore, the women with diabetes are at risk of delayed lactogenesis ll, which aBME is thought to decrease^12^. For these women aBME seems beneficial due to the possibility of having a supply of expressed milk prior to birth and it might promote breastfeeding establishment. Additionally, aBME could also support the availability of mother’s own milk directly after preterm birth. However, the safety of aBME in women at risk of preterm birth is yet to be studied. More knowledge on the mother’s experience with aBME is warranted^13,14^.

Safety of aBME performed before 36 weeks of gestation in healthy women is not weel-documented and a description of the bodily experience is unknown. The importance of adding more research in relation to aBME and breastfeeding establishment can contribute to more knowledge in supporting women at risk of breastfeeding establishment, and thereby possibly prolong the duration of breastfeeding.

Investigating whether healthy pregnant women can benefit from breastmilk expression during pregnancy can help to understand some mechanisms in the process of breastfeeding establishment. This will contribute new knowledge about whether aBME can lead to increased readiness for women in the early stages of breastfeeding, and thus affect the duration of breastfeeding.

This study aims to examine women’s bodily experience of aBME.

## METHODS

### Study design and setting

A qualitative interview study was conducted to answer the aim of this study. A phenomenological-hermeneutic approach was used to examine the subjective experience of the women and to stay close to the descriptions of their experiences with aBME. Thematic analysis was used for analysis. This study was part of a larger research protocol (clinicaltrials.gov NCT05516199). The study was conducted at Odense University Hospital in Denmark, providing highly specialized treatment and care for pregnant women. aBME is only recommended as a routine procedure in Denmark for women with diabetes in pregnancy from 36 weeks of pregnancy.

### Research team and reflexivity

The study was carried out by a multidisciplinary research team with a neonatal nurse, one midwife, two neonatologists and one obstetrician. The interviews were conducted by the midwife (first author) and analyzed together with the neonatal nurse (last author). Prior to this study none of the researchers in the study had experience with aBME as an intervention. All the participating women had met the interviewer prior to the interviews for a breastfeeding consultation when starting aBME at 34 weeks of pregnancy.

### Participants

The women were recruited from the intervention group of the randomized controlled pilot study Express-MOM (NCT05516199), investigating safety and outcomes for mother and infant, when performing aBME from 34 weeks of gestation. Women were included in the Express-MOM study from 31 August 2022 to 24 May 2023 through midwife consultations and an information video on the hospitals Facebook page. Participants were healthy nulliparous Danish-speaking women with no major chronic or pregnancy-related diseases, no prior breast surgery, and body mass index ≤27 kg/m^2^, who hoped for giving birth at Odense University Hospital and planning exclusive breastfeeding for six months. All 60 women participating in the randomized controlled pilot study were invited to a breastfeeding consultation with a trained midwife (the first author) prior to the intervention. The consultation consisted of both theoretical information, and practical exercises with a doll that was used to demonstrate different nursing positions. All the women were advised to invite their partners to participate in the breastfeeding consultation. The women from the intervention group were during the breastfeeding consultation instructed on how to hand express, and how to store any milk collected.

The 30 women in the intervention group were instructed to hand stimulate each breast 2 times a day for 5 minutes and store and freeze any expressed milk in small containers provided for the purpose. From the start of the intervention until eight weeks after birth, short weekly questionnaires were sent to the women using an app dedicated for the purpose. Questions before birth concerned gestational age at birth, and the intervention group received questions covering number of weekly breastmilk expressions and the amount of expressed milk if any. After birth, questions concerned exclusive breastfeeding and any supplementary formula feeding. Exclusive breastfeeding was defined according to the World Health Organization meaning that the infant received no other liquids than the mother’s own milk^1^. Women from the intervention group were recruited consecutively for in-depth semi-structured interviews at the midwife consultation, and all seven women that were asked to participate in the interview, attended. No women refused to participate in an interview.

### Data collection

A semi-structured interview guide was developed by the first and last author, and contained themes related to the aim of the study. Questions regarded thoughts of attending breastfeeding consultation, starting aBME, bodily experience of performing aBME and whether performing aBME bodily affects the early stages of breastfeeding. The interview guide was pilot-tested on two midwives who work in the delivery ward at Odense University Hospital and recently gave birth. The testing was to ensure that the questions were understandable. Feedback concerned the meaning of words, especially about body image, which led to changing words incorporated into the final interview guide. The first author conducted in-depth semi-structured face-to-face interviews in the participant’s home from December 2022 to March 2023. Data saturation was reached after six interviews, but another interview was conducted to ensure the data saturation. The interviews were audio recorded and verbatim transcribed. Field notes were not taken. The interview transcripts were in the Danish language and quotes supporting the analysis were analyzed into English. The participating women did not read the interview transcripts for their review. All the women who were included for interview participated.

### Analysis

Interview data were analyzed using Braun and Clarke's thematic analysis, which consists of 6 steps: 1) Reading and re-reading the dataset to get familiarized with the data; 2) Generating initial codes; 3) The process of searching for themes begins; 4) Reviewing themes; 5) Defining and naming themes; and 6) Writing the report^15^.

During the second step of the analysis, the first (SBB) and last author (KGH) individually extracted codes from the interview transcripts to achieve several perspectives of the data. Finally, the codes were triangulated between SBB and KGH to reach consensus. In the process of defining and naming themes, a table supported organization of the codes into subthemes. Finally, the themes were identified and discussed between SBB and KGH to ensure consensus on the themes. All authors approved the themes. The participants did not provide feedback on the findings.

### Ethics

The participants received both oral and written information and signed a written consent to participate in the study. Ethical approval was obtained through the Ethical Committee of Southern Denmark (project number: 22/2533; 22 November 2021) and by the Data Protection Agency at the Region of Southern Denmark (21/59493; 8 November 2021).

## RESULTS

Seven women participated in the interviews which took place 2–4 weeks after birth and the average duration of an interview was 41 minutes (27–62 minutes). The participants mean age was 28.7 years. Six of the mothers had a vaginal delivery (86%) and all the mothers were exclusive breastfeeding at the time of the interview (Table 1).

**Table 1:** Mothers’ baseline information from a qualitative interview study exploring women’s bodily experience of antennal breastmilk expression, Denmark, 2024 (N=7)

*Characteristics*	*n (%)*
**Age** (years), mean (95% CI)	28.7 (27.1–30.3)
**Education level** (years)	
High (≥5)	4 (57)
Middle (≥3.5)	3 (43)
**Vaginal delivery**	6 (86)
**Week since birth at interview,** mean (range)	3.29 (2–4)
**Antepartum breastfeeding consultation**	
Have been breastfed as a child	6 (86)
Received other breastfeeding preparation	7 (100)
Partner participated in the Express-MOM consultation	7 (100)
Cohabiting with the baby’s father	7 (100)
**Women who were able to express milk during pregnancy**	6 (86)
**Exclusively feeding with mothers’ milk until interview**	7 (100)
**Breastfeeding postpartum**	
Exclusive breastfeeding first 24 hours after birth Exclusive breastfeeding at interview*	7 (100) 7 (100)

* Definition of exclusively breastfeeding based on World Health Organization (WHO)^1^: No other food or liquids than the mother’s milk, including water, are provided to the infant.

**Figure 1:**
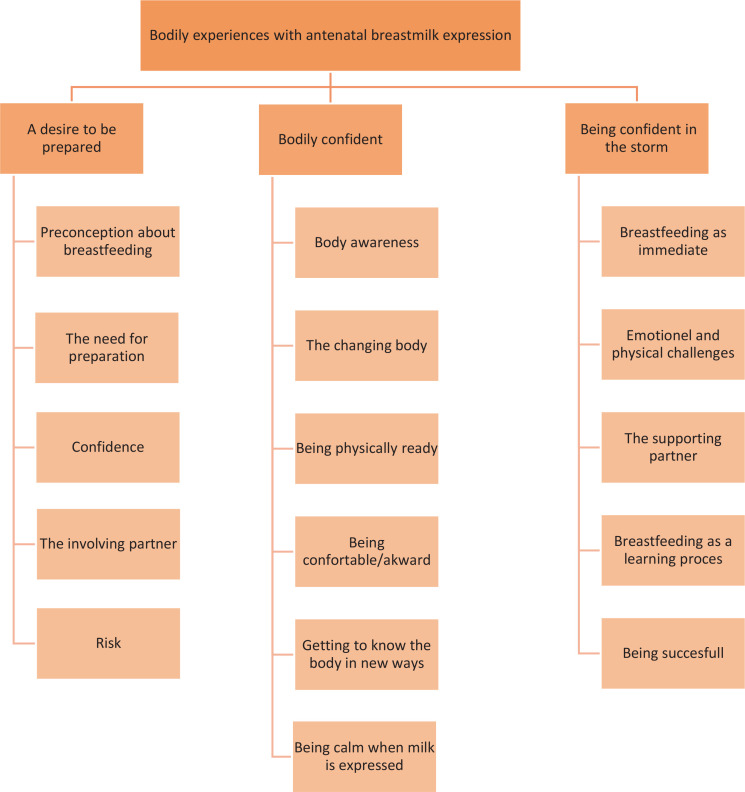
Themes and subthemes from interviews with women after birth, a qualitative interview study exploring women’s bodily experience of antennal breastmilk expression, Denmark, 2024 (N=7)

Analyses of the interview transcripts revealed three themes: 1) A desire to be prepared, 2) Bodily confident, and 3) Being confident in the storm (Figure 1), based on five, six, and five subthemes respectively.

### A desire to be prepared

All women reported that they participated in the Express-MOM study to prepare themselves for breastfeeding and to gain knowledge, and thereby increase their odds of accomplishing establishment of exclusive breastfeeding. They perceived breastfeeding as the best nutrition for their infants, and many also described how they saw breastfeeding as a way of getting intimacy and close contact with their infants:

*‘I wanted to succeed with breastfeeding because of the relationship and intimacy I would get with my baby.’* (MOM ID6)

Many had preconceptions of breastfeeding as being difficult to establish:

*‘Throughout the pregnancy I have had thoughts about what breastfeeding would be like, and how difficult it would be.’* (MOM ID2)

Some described stories from acquaintances for whom breastfeeding establishment was difficult but succeeded because they sought help through self-paid courses. Throughout the pregnancy, some women felt that preparation for breastfeeding was deprioritized by the healthcare system. They received short oral information and a folder or an online course from healthcare providers. The breastfeeding consultation in relation to the Express-MOM study provided the women with theoretical and practical support. Some women described how the consultation provided feelings of confidence:

*‘I got much calmer after attending the breastfeeding consultation and I was absolutely sure that I was going to be able to breastfeed.’* (MOM ID6)

Others describe how the breastfeeding consultation had influenced them to be able to see several perspectives on what to expect from the breastfeeding establishment. All the women were excited about the practical exercises where they tried different nursing positions with the doll:

*‘It gave me a lot because I learn best while having hands-on.’* (MOM ID3)

Furthermore, the women experienced how practical exercises provided their partners being more involved, by connecting the theoretical knowledge to practical skills:

*‘It’s just been so concrete for my partner, instead of seeing some online video on how to breastfeed.’* (MOM ID5)

Some women experienced how the offer of one-to-one private consultation left them and their partner open to ask any questions:

*‘I felt open to ask questions about my breast and nipple shape.’* (MOMID2)

The women in this study did not think much of the potential risk of participating. They felt well-informed prior to performing aBME and generally believed that breastmilk expression was not associated with preterm birth:

*‘I didn’t think about the risk, more about what the advantages would be.’* (MOM ID1)

Some also mentioned how an uncomplicated pregnancy affected their belief in trusting their body:

*‘I didn’t think about the risk, I trusted my body.’* (MOM ID6)

### Bodily confident

The women’s body image was overall related to them being comfortable in their bodies before pregnancy, yet with focus on having a healthy strong body with daily workouts and healthy food. Some women shared thoughts of being aware of how body image had affected their self-perception throughout their lives:

*‘Body image has been a big part of my life. I am aware that it has filled a lot.’* (MOM ID5)

During pregnancy, all the women embraced their bodily changes and were excited about the physical ability of their bodies. Some shared feelings about being proud of their faithful body:

*‘I just thought it was something beautiful and fine, and I was looking forward to see how my body reacted.’* (MOM ID7)

Others worried that the bodily changes during pregnancy would have taken up more space, but did not. Instead, thoughts about the body after being pregnant occupied some of the women:

*‘I think what filled me the most was the one about how my body will be afterward.’* (MOM ID4)

The women experienced that aBME made them physically ready for breastfeeding. Prior to the study the majority of the women were not familiar with the possibility of expressing milk in pregnancy:

*‘I thought it was something you did when the baby was born.’* (MOM ID2)

Because of the wish to prepare themselves for breastfeeding, the women hoped to be randomized to the intervention group, when they signed up for the study. They thought it would increase physical readiness and help them understand what breastfeeding would be like. Few of the women felt it was feasible to perform aBME twice daily due to being time consuming. However, most of the women were eager to get started:

*‘Great now we’re going to get started, now we will see what my body and breasts can do.’* (MOM ID3)

Different perspectives emerged regarding whether it felt natural to perform aBME. Some women described how they felt uncomfortable because they did not associate their breasts with food yet:

*‘To sit and massage the breast and try to get some milk out, that felt very un-sexual.’* (MOM ID4)

While others found it to be a natural thing to do:

*‘I had a purpose by doing it, it has been some kind of natural preparation.’* (MOM ID7)

Almost all the women described how they gained a different understanding of their body after performing aBME. Some described how they reflected breast function when performing aBME:

*‘One’s breasts starts as something feminine, and later it becomes something more sexual, and then it ends up being food for one’s infant.’* (MOMID7)

Others viewed feelings of physically preparing the breast while performing aBME:

*‘You get a better understanding of the breast, you feel them, and thereby get more attached to them.’* (MOM ID1)

Many of the participants experienced an increased volume of milk towards the end of pregnancy. Being able to express milk increased a sense of calmness and provided confidence to succeed with breastfeeding:

*‘It gave me a feeling of being able to successfully breastfeed. I knew I had milk, and that made me believe that I was going to be able to handle it physically.’* (MOM ID2)

The women who were able to express and collect milk during pregnancy were proud of their bodies, but they also had expectations of how their bodies should perform:

*‘I wanted to perform - the more milk, the better.’* (MOM ID6)

If the body did not fulfill the expectations, the women worried about why the milk volume in some periods did not increase:

*‘Around my due date the amount of milk fluctuated, which made me feel like my level of oxytocin was low.’* (MOM ID5)

The women were asked to reflect on whether they thought it would have affected them if they were not able to express any milk. All the women that were able to express milk stated that they would probably have been more uncertain and sensed that their body would not perform as they had hoped.

### Being confident in the storm

Prior to the breastfeeding establishment, the women felt confident and ready to breastfeed:

*‘I felt confident about how to latch my baby to the breast.’* (MOM ID5)

Being physically ready for breastfeeding establishment, strengthened their confidence to succeed with breastfeeding. Some also described how they felt good with the first breastfeeding experience, being able to act immediately:

*‘It was so natural to breastfeed.’* (MOM ID5)

Even though the first breastfeeding experience felt natural and without complications, some of the women felt both emotionally and physically challenged in the establishment of breastfeeding:

*‘It has been hard, frustrating, interesting, and exciting.’* (MOM ID2)

Uncertainties occurred when breastfeeding was difficult, in situations where it was hard to get the baby to latch on, if the women were having pain while breastfeeding, or if the baby was crying:

*‘I felt so sure about being ready for breastfeeding, but it hasn’t been easy, so the feeling is gone for now.’* (MOM ID4)

For some, emotional doubts also occurred:

*‘My challenges have been mental, this feeling of being the only one having breastfeeding problems made me think, that maybe it was me.’* (MOM ID 2).

Also, the time spent on breastfeeding surprised some of the women, leaving them with feelings of getting no time for themselves:

*‘You don’t have time to do other things, so everything around you and your own needs is put aside.’* (MOM ID3)

The preparation for breastfeeding was a shared project between the woman and her partner, and the common understanding they both gained of what it required to establish breastfeeding, made them see breastfeeding as a shared project. With support from their partners, the women strengthened their belief in themselves, which reinforced their belief in their ability to succeed in breastfeeding:

*‘We were together about breastfeeding.’* (MOM ID1)

Overall, the women felt calm during the initial breastfeeding, but it was emotionally challenging for some of the women when breastfeeding establishment was difficult. The women felt emotionally ready and confident before initiating breastfeeding, but still, some women were surprised by the feelings related to becoming a mother and at the same time establishing breastfeeding. Performing aBME during pregnancy made most of the women relaxed by knowing that they had enough milk for their baby:

*‘I felt calm knowing I had enough milk.’* (MOMID6)

Additionally, the majority of women recognized that breastfeeding is a learning process:

*‘You need to get to know each other and learn how to interpret the signals.’* (MOM ID4)

They felt reassured knowing that breastfeeding would get better in time:

*‘It is getting better each day. Perhaps in one month it will be easier than now. So, I try to think that it will be better.’* (MOM ID1)

They were all eager to succeed and expected that they, both emotionally as well as physically, would be able to continue breastfeeding despite the challenges they experienced in the early breastfeeding establishment:

*‘It has been a rollercoaster, but I am holding on, I want to succeed.’* (MOM5)

Despite the struggle and emotional challenges during the early period of breastfeeding establishment, all the women succeeded with exclusive breastfeeding the first 24 hours after birth and up to the time of the interview.

The women felt that aBME had provided them skills to establish successful breastfeeding:

*‘I have used the techniques from the hand stimulation.’* (MOM4)

The women were so positive of the benefits of aBME and shared thoughts about performing aBME in another pregnancy, regardless of the expressed milk volume they were able to collect. Some also encouraged other pregnant women to participate in the study, because they felt that aBME had helped themselves.

## DISCUSSION

This study found that the participating women had a deep desire to prepare themselves for breastfeeding and were highly motivated to perform aBME. Having the experience of expressed milk from aBME made the women bodily confident and perceive that their body was physically ready for breastfeeding. When performing aBME, the women experienced gaining a better understanding of the function of the breast which they used after birth. This meant that the women felt it easier to handle and shape the breast for breastfeeding.

To our knowledge, this is the first study investigating bodily experiences of aBME from 34 weeks of gestation among nulliparous healthy women with low-risk pregnancies.

Although almost all women in this study experienced some challenges when establishing breastfeeding, they all felt convinced that they would eventually succeed due to the knowledge they had gained during pregnancy (in theory and practice) and the body awareness they felt they achieved through aBME. Consistent with previous studies^16,15^, we found that aBME gave women feelings of confidence in achieving breastfeeding success. According to Nilsson et al.^17^, negative breastfeeding experiences through the first week after birth are crucial for women’s breastfeeding self-efficacy one week following birth. Through aBME, the women felt provided with tools to use when latching the baby on the breast, resulting in early positive breastfeeding experiences. These early positive breastfeeding experiences may have increased the women’s confidence towards breastfeeding.

Yet women have different motives to participate in studies investigating aBME. While for women with diabetes, the primary goal was to increase infants’ health and reduce the need for formula feeding after birth^13,15,18^. The women in this study aimed to achieve knowledge and confidence through aBME, and they discussed feelings of being positive when their bodies were able to increase milk volume during the period of performing aBME. Some got affected when the volume of milk fluctuated, which influenced their beliefs in their body performance. This emphasizes the importance of clarifying information to the women, that collecting milk was not a goal but merely an extra benefit, and that milk volume may vary from day to day, and not necessarily continuous increase until birth.

The women’s perspective on body image and their ability to fulfill their body expectations may have affected their perspective on the desire to succeed in breastfeeding. This was also found in the study by Gillen et al.^8^ who presented perspectives on how positive body image may strengthen women’s commitment to establish breastfeeding. Performing aBME made the women more connected to their breasts, which might increase the women’s confidence in their bodies and thereby breastfeeding establishment. This is similar to findings by Brisbane et al.^16^ on how women gained new knowledge of their breasts by performing aBME.

In addition, the women in this study reported how they gained an awareness of functions of the breast earlier than expected due to performing aBME. Reflecting the journey of the breast, starting with being something feminine, then sexual, and lastly providing food for their infants. This changing breast function was for some of the women challenging, because seeing the breast as a nutrient source was related to having the infant latching on the breast. Women realized that the transition of the breast functions may contribute to achieving better convenience in breastfeeding establishment. This is also mentioned by Broers et al.^9^ who find that the establishment of the relationship between body image and breastfeeding can increase women’s comfort while breastfeeding.

In this study, aBME was an intervention that was provided together with a breastfeeding consultation between 33 and 34 weeks of pregnancy. Studies investigating succesful breastfeeding interventions find divergent results regarding what is effective when preparing women for breastfeeding. Skouteris et al.^18^ find that the most successful breastfeeding intervention, features a supportive or educational approach for at least four months after birth. This is achieved by using one-on-one breastfeeding guidance during hospital stay, telephone calls or access to chat through website or text messages from a lactation specialist. All of the interviewed women had hospital admission after birth for at least 24 hours due to complications after birth or on maternal request. However, during admission the women experienced not feeling sufficiently supported in breastfeeding establishment. Instead, they relied on the knowledge and tools learned from the breastfeeding consultation and their aBME experiences obtained during pregnancy. It is important to mention that aBME is an intervention before birth and that the study of Skouteris et al.^18^ find that interventions providing long-term postpartum support are the most effective in increasing breastfeeding rate. Findings from our study indicate that aBME can be a supportive intervention to prepare women for breastfeeding and support exclusive breastfeeding in the first week after birth, maybe due to the acquired knowledge and trust in how to position their baby (practical training) and how to shape their nipple to get the baby to latch on (aBME). This indicates that the women felt supported by the practical training in combination with aBME. These findings are consistent with Kehinde et al.^19^ who claim that by increasing women’s knowledge together with holding a positive approach towards breastfeeding, increases women’s initiation and duration of breastfeeding. However, Moorhead et al.^20^ have, through a randomized controlled trial, studied the effect of aBME in diabetic women and found no association with any or exclusive breastfeeding. aBME is still a relatively new intervention and more research of its potential impact is needed.

In this study, all the women’s partners participated in the breastfeeding consultation. The women experienced that their partner’s participation increased his knowledge on breastfeeding and encouraged the women to perform aBME every day. Although the control group also received theoretical information and practical teaching at the breastfeeding consultation, the focus on the breast through aBME might have increased the father’s focus on breastfeeding and thus encouraged more conversations about breastfeeding during pregnancy. This made the women feel that breastfeeding was a joint project. According to Namir et al.^21^, fathers’ involvement and support influences the decision to breastfeed, and their engagement positively affects breastfeeding initiation and duration. Practicing aBME during pregnancy as a joint project and may support the couple’s collaboration on breastfeeding after birth^22^.

During the first couple of weeks after birth, most of the women in this study struggled with their new role as a mother and at the same time having to learn how to breastfeed. Kronborg et al.^23^ describe similar findings about learning how to become a mother and breastfeeding at the same time; however, at the same time the women felt calm knowing that breastfeeding is a learning process. In addition, the women did benefit from not having to worry about whether they were able to produce enough milk for their infants. This led them to feel calm when establishing breastfeeding. Other studies^23,24^ report that this is one of the major worries women have when establishing breastfeeding. Further, this is also a major cause for early breastfeeding cessation^25,26^. This might indicate that women that are able to express milk during pregnancy might be more confident with their ability to provide a sufficient milk supply. However, it still remains unclear how aBME affects body image and breastfeeding self-efficacy when the woman is not able to express milk.

The results from this study and the outcome study of Express-MOM have shown that breastmilk can be expressed and thereby be available for the infant directly after birth. Making mothers own milk available after birth has great potential especially for preterm infants. It is recommended to feed preterm infants with mother’s own milk as soon as possible after birth, but mother’s own milk is not always available. The findings from the Express-MOM study indicate that aBME supports the availability of mother’s own milk after birth and supports the women in establishing breastfeeding. More research that focuses on the safety of aBME and availability of milk in high-risk pregnancies is needed. Thereby, it might be possible to make mothers own milk available to preterm born infants immediately after birth. Furthermore, maybe increase women's confidence in the vulnerable breastfeeding establishment.

### Limitations

This study was carried out in a group of highly educated women in a country with focus on breastfeeding and high rates of breastfeeding initiation^27-30^. A limitation of this study is the small sample of participants. Further, the participating women were a homogeneous group of first-time high educated new mothers. Data saturation of the interviews was reached after six interviews in regard to experience of aBME and the following breastfeeding establishment. A limitation of the study is the limited diversity in the women’s experience of not being able to express milk, as it only happened to one woman. The findings regarding this issue must therefore be interpreted with caution. The interviews were conducted between 2 and 4 weeks after birth, which might have been too soon due to some of the women still having challenges with breastfeeding establishment. However, all the women emphasized that breastfeeding establishment is a learning process that will take time, and they perceived that no preparation could have avoided that process. The first author was responsible for the breastfeeding consultation and carried out the interviews. Potential conflicts of a dual role of the researcher have been raised^31^. This previously formed relationship before the interviews may have influenced the women’s responses positive to the questions raised. The first and last author reflected on this subject prior to the interviews to support the first author’s awareness of her role, especially during the interviews. However, it must be considered that the analysis was triangulated, meaning the first author with a dual role was not alone responsible of the interpretation of the interview transcripts. Another reflection is that the interviews were conducted in Danish and quotes translated subsequently for this article, after the data were analyzed.

It must also be considered that the women participating in this study received two interventions: a breastfeeding consultation and aBME. The increased confidence for breastfeeding that the women felt cannot solely be related to aBME but must be seen in a combination of the two interventions. To support the transparency of the study the ‘Consolidated criteria for reporting qualitative research’ (COREQ) checklist was used for reporting^32^.

## CONCLUSIONS

Conducting aBME from 34 weeks of pregnancy for healthy women may increase their bodily awareness and seems to prepare the women for breastfeeding. The findings highlight the importance of investigating whether aBME is safe for women at risk of preterm birth and whether it supports exclusive breastfeeding following high-risk pregnancies.

## Data Availability

The data supporting this research are available from the authors on reasonable request.
